# A Spatial-Temporal Approach Based on Antenna Array for GNSS Anti-Spoofing

**DOI:** 10.3390/s21030929

**Published:** 2021-01-30

**Authors:** Yuqing Zhao, Feng Shen, Guanghui Xu, Guochen Wang

**Affiliations:** 1 School of Instrumentation Science and Engineering, Harbin Institute of Technology, Harbin 150001, China; zhaoyuqing@hit.edu.cn (Y.Z.); fshen@hit.edu.cn (F.S.); 2 State Key Laboratory of Satellite Navigation System and Equipment Technology, Shijiazhuang 050081, China; xghdh407@126.com

**Keywords:** global navigation satellite system (GNSS), spoofing detection, antenna array, eigen space, power comparison, oblique projection, cross-correlation monitoring

## Abstract

The presence of spoofing signals poses a significant threat to global navigation satellite system (GNSS)-based positioning applications, as it could cause a malfunction of the positioning service. Therefore, the main objective of this paper is to present a spatial-temporal technique that enables GNSS receivers to reliably detect and suppress spoofing. The technique, which is based on antenna array, can be divided into two consecutive stages. In the first stage, an improved eigen space spectrum is constructed for direction of arrival (DOA) estimation. To this end, a signal preprocessing scheme is provided to solve the signal model mismatch in the DOA estimation for navigation signals. In the second stage, we design an optimization problem for power estimation with the estimated DOA as support information. After that, the spoofing detection is achieved by combining power comparison and cross-correlation monitoring. Finally, we enhance the genuine signals by beamforming while the subspace oblique projection is used to suppress spoofing. The proposed technique does not depend on external hardware and can be readily implemented on raw digital baseband signal before the despreading of GNSS receivers. Crucially, the low-power spoofing attack and multipath can be distinguished and mitigated by this technique. The estimated DOA and power are both beneficial for subsequent spoofing localization. The simulation results demonstrate the effectiveness of our method.

## 1. Introduction

GNSS receivers are tremendously used in navigation and timing applications because of their high accuracy, all-weather, and global operation. However, weak satellite signals, together with potential interference including jamming and spoofing, make GNSS-dependent infrastructures at a serious risk [1,2]. The jamming is aim to degrade the carrier to noise ratio of the victim receiver and prevent it from performing signal acquisition and tracking [3]. Fortunately, it is easy to detect and mitigate jamming due to the high power and different structure from genuine GNSS signals [4,5,6,7]. As an effective anti-jamming technology, spatial processing based on an antenna array can suppress the interference by forming nulls toward the direction of jamming [7]. Compared with a typical jammer, a spoofer that counterfeits GNSS signals or retransmits genuine signals is more sinister, which will lead to a hazardously misleading position or time [8].

As a response to spoofing attack, several detection techniques have been proposed [9,10,11,12,13,14,15,16,17,18,19]. The main categories are identified: cryptographic GNSS anti-spoofing [9], methods using external verification sources [10,11], and approaches exploiting signal features [12,13,14,15,16,17,18,19]. The encryption authentication based anti-spoofing technique is the most effective but is hard to implement at the present stage [9]. This is because this authentication technique requires GNSS signals to be designed to support encryption. Likewise, methods using external verification sources [10,11] need to be equipped with inertial sensors or vision sensors, which is impractical in applications since the receiver may not be able to afford the additional size, cost, and weight. It seems that approaches based on signal features are effective and practicable, but the approach that exploits only one signal feature may suffer an unbearable false alarm rate. For example, in terms of power monitoring technique, the authors in [15] point out that the challenge of the spoofing detection method relying only on power characteristics is that the jammer or common personal privacy devices (PDDs) in urban areas may trigger the spoofing detector. Furthermore, it is demonstrated in [16] that the spoofer whose power is 0.4 dB higher than the corresponding satellite signal can reliably spoof the target receiver, which is difficult to detect by power monitoring. For the correlation function distortion spoofing detection technique in [17,18], the multipath and the spoofing are difficult to distinguish. Among the different spoofing detection approaches exploiting signal features, the direction of arrival (DOA) defense takes advantage of the assumption that the satellites signals come from different directions while counterfeit signals are broadcast from a single source, which is considered as one of the most effective because it does not require encryption function and other external verification infrastructures [19].

However, merely detecting spoofing is not enough, the anti-spoofing techniques eventually aim to suppress the spoofing threat and recover the timing and positioning capabilities [20]. The spatial processing technology based on the antenna array can not only detect spoofing but also neutralize the spoofing threat, which can be realized in the two stages of predespreading and postdespreading. The anti-spoofing technique in [21] belongs to the predespreading method, where the eigenvector corresponding to the largest eigenvalue is regarded as the spoofing subspace, which can be obtained by the spatial correlation matrix, and then the spoofing is suppressed by subspace projection. Although the complexity of the method is lower, it is only suitable for spoofing with significantly higher power than satellite, which means this method does not work well when low-power spoofing signals are transmitted. The postdespreading methods in [22,23] perform correlation and accumulation processing on each antenna sample, then the DOA estimation is intended to distinguish between spoofing and authentic signals. The beamforming is utilized to suppress the spoofing and protect the authentic satellite signals [24]. However, since the receiver requires a large number of correlators, the high computational complexity makes it difficult to apply in practice.

In [25] our antenna array approach to detection of GNSS spoofing is presented, which can effectively detect the existence of the spoofing signal even in the presence of multipath. However, it focused on spoofing detection and did not provide further solutions for spoofing and multipath. In addition, the accuracy of the source estimation was affected by multiple correlated signal sources (GNSS spoofing, multipath, and authentic signals), which makes it a challenge to detect low-power spoofing.This paper, a significant improvement and extension of our work in [25], makes better performance in DOA estimation, power estimation, and anti-spoofing. Specifically, an improved eigen spatial spectrum for DOA estimation is formulated by introducing propagation method (PM), where a signal preprocessing is proposed to overcome the impact of low SNR and multiple correlated sources, which are utilized to determine the incident directions of all sources. After that, an optimization problem based on the covariance matrix is designed to obtain the signal power corresponding to each spatial incident angle, in which the estimated DOA is taken as a priori. According to the estimated DOA, we separate the incident signals and divide them into different spatial channels. Then we perform cross-correlation calculations arbitrarily on two different spatial channels and monitor the number of cross-correlation peaks. The power estimation results and cross-correlation results are combined to authenticate spoofing and multipath, which can overcome the shortcomings of a single detection method. Finally, we provide an interference suppression scheme, in which the spoofing and multipath can be suppressed by oblique projection while each genuine signal is enhanced by beamforming.

The main contributions of this paper can be summarized as follows:

(1) We present a novel technique based on antenna array for GNSS anti-spoofing, which can not only distinguish low-power spoofing from multipath but also provides advanced signal processing methods for multipath and spoofing mitigation.
(2) The DOA and power offered by the improved spatial spectrum estimation and enhanced power estimation can be used as support for the subsequent spoofing localization.
(3) All operations are based on the baseband samples, without the need to perform despreading processing on the receiver, which avoids the acquisition and tracking of the receiver and thus does not bring additional computational complexity to the GNSS receivers.

The rest of this paper is organized as follows. In Section 2, the received signal model based on uniform linear array (ULA) is introduced. Then, the GNSS spoofing detection and mitigation scheme is presented in Section 3. In Section 4, the performance of the provided DOA estimation and power estimation are evaluated through simulation results. Furthermore, more simulation results in three application scenarios validate the effectiveness of the proposed anti-spoofing scheme. Section 5 concludes this paper.

## 2. Signal Model

It is worth noting that current signal models based on array antenna focus on line of sight reception models for authentic signals and spoofing. However, in practice, GNSS receivers are usually subject to multipath reflections. Due to the similarity of multipath signals with authentic signals and spoofing, spoofing is more difficult to detect, especially in the early stage of the spoofing. Therefore, in this paper, we consider that the authentic signal, multipath and spoofing arrive at an *M*-element antenna array. Without loss of generality we assume that all the counterfeit signals are transmitted by a single-antenna, whether it is receiver-based spoofing or generator-based spoofing. The received signal is first discretized by the sampling frequency $f_{s}$, and the resulting signals constitute the M×1 array signal vector as follows:

(1) $$x\left(nT_{s}\right)=S^{a}\left(nT_{s}\right)+S^{s}\left(nT_{s}\right)+S^{m}\left(nT_{s}\right)+V\left(nT_{s}\right)$$

where $V(nT_{s})$ is the complex addictive white Gaussian noise vector and $T_{s}=1/f_{s}$ is the sampling interval. The authentic signal $S^{a}\left(nT_{s}\right)=\sum_{i=1}^{N^{a}}a\left(\theta_{i}\right)s_{i}\left(nT_{s}\right)$, the spoofing $S^{s}\left(nT_{s}\right)=\sum_{i=1}^{N^{s}}a\left(\theta_{s}\right)s_{i}^{\prime }\left(nT_{s}\right)$ and the multipath $S^{m}\left(nT_{s}\right)=\sum_{q=1}^{N^{m}}a\left(\theta_{q}\right)s_{q}^{\prime \prime }\left(nT_{s}\right)$. In the above equation, $N^{a}$, $N^{m}$ and $N^{s}$ represent the number of genuine satellite signals, multipath, and spoofing, respectively. The symbols $a(\theta_{i})$, $a(\theta_{q})$, and $a(\theta_{s})$ denote the ULA steering vectors of the *i*-th genuine signals, the *q*-th multipath and the spoofing respectively. Furthermore, $s_{i}\left(n\right)$ means the *i*-th authentic satellite signal, $s_{i}^{\prime }\left(n\right)$ denotes the *i*-th spoofing signal, and $s_{q}^{\prime \prime }\left(n\right)$ is the *q*-th multipath. The power ratio of $s_{i}^{\prime }\left(n\right)$ and $s_{i}\left(n\right)$ is usually greater than 1 during the takeover of the target GNSS receiver, while the power ratio of $s_{q}^{\prime \prime }\left(n\right)$ and $s_{i}\left(n\right)$ is less than 1 in general.

Since the ULA is utilized, for the incident angle $\theta_{k}$, the steering vector can be expressed as

(2) $$a\left(\theta_{k}\right)={\left\lceil 1,e^{-j2\pi d\sin\theta_{k}/\lambda },\ldots ,e^{-j2\pi d\left(M-1\right)\sin\theta_{k}/\lambda }\right\rceil }^{T}$$

where the parameter *d* represents the distance between two adjacent array elements, which is taken as λ/2 in this paper. The symbol λ=c/f denotes the signal wavelength of GPS $L_{1}$, where *c* is the speed of light and *f* is equal to 1575.42 MHz. In our signal model, the ULA consists of 16 omnidirectional sensors.

## 3. Proposed Anti-Spoofing Scheme

**Figure 1:**
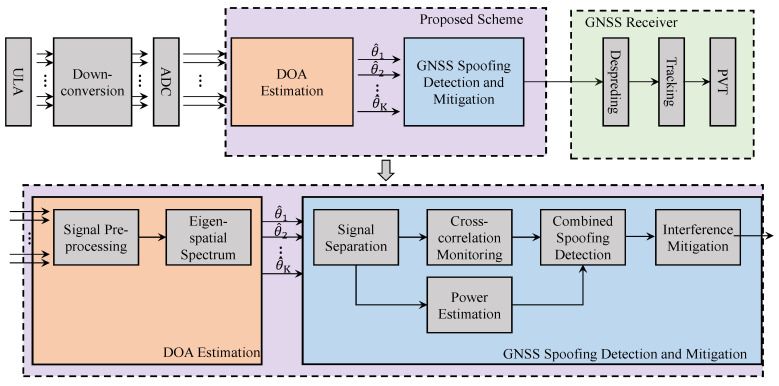
Block diagram of the proposed anti-spoofing scheme.

The block diagram of the suggested anti-spoofing scheme is shown in Figure 1. It is implemented in two stages, namely DOA estimation and spoofing detection and mitigation. In the DOA estimation module, we first design a preprocessing method suitable for signal model, where the GNSS self-coherent characteristic is used to suppress the noise component. Futhermore, the forward/backward spatial smoothing technique is performed to reduce the correlation between the GNSS spoofing, multipath, and authentic signal. Then, an eigen-spatial spectrum based on PM is proposed to obtain DOAs of all incident signals. The second stage of this technology is spoofing detection and mitigation, which mainly contains four steps: (1) Power calculation and comparison, (2) Spatial channel separation, (3) Time domain cross-correlation peak monitoring, (4) Spoofing mitigation. All operations of the proposed technology are performed on the raw digital baseband signal without depending on external hardware. The following sections provide detailed information on these stages.

### 3.1. DOA Estimation

We propose an improved eigen-space DOA estimation algorithm for spoofing, multipath, and genuine satellite signals in this subsection, where the signal preprocessing algorithm is performed to solve the model mismatch of the DOA estimation algorithm caused by low signal to noise ratio (SNR) and related signal sources. Furthermore, a new eigen-spatial spectrum can be constructed by introducing the PM without eigen-decomposition, and then the DOAs of the signal sources can be obtained by searching the peaks of the spatial spectrum.

#### 3.1.1. Signal Preprocessing

On the one hand, the genuine GNSS signal is a typical weak signal whose power is 20–30 dB lower than the noise. In most GNSS applications, to be more effective, a spoofer might transmit several PRN signals with consistent features. Therefore, the presence of multiple spoofing signals can considerably increase the power content of received signals within the bandwidth. However, since the spoofing signals still below the noise level, it is very challenging for GNSS receiver to detect and separate multiple spoofing signals from the received raw signal. To address this problem, the characteristics of GNSS signals are fully exploited in this paper. It is well known that counterfeit signals also have periodic structures similar to the authentic signals. In addition, their chip rate samples, which is separated by integer multiples of spreading gain, have strong self-coherence. Taking GPS as an example, the C/A code sequence is repeated 20 times in each navigation symbol, and the spreading gain is G = 1023. For simplicity, we use *n* to represent the *n*-th sampling time point *n*$T_{s}$, then the reference data can be formulated as

(3) $$\begin{array}{cc} x(n-jG) & =S^{a}\left(n-jG\right)+S^{s}\left(n-jG\right)+S^{m}\left(n-jG\right)+V\left(n-jG\right)\\ & =S^{a}\left(n\right)+S^{s}\left(n\right)+S^{m}\left(n\right)+V\left(n-jG\right) \end{array}$$

in which, *j* is a positive integer and j∈[1,20), the distance between the corresponding sample in x(n), and x(n−jG) is equal to the *jG* chips. The covariance matrix of the received signal can be obtained by

(4) $$R_{xx}=E\left\{x\left(n\right)x^{H}\left(n\right)\right\}={\mathrm{AP}}_{PRNs}A^{H}+\sigma_{V}^{2}I$$

where $A={\left[a\left(\theta_{1}\right)\ldots a\left(\theta_{L}\right),a\left(\theta_{s}\right)\right]}_{M\times (L+1)}$ denotes the ULA steering matrix of the PRN signals, $L=N^{a}+N^{m}$. The matrix $P_{PRNs}=\mathrm{diag}({\left[\sigma_{1}^{2},\sigma_{2}^{2},\cdots ,\sigma_{K}^{2}\right]}^{T})$ is a diagonal matrix, whose diagonal element $\sigma_{k}^{2}$ represents the power of the *k*-th signal source, and K=L+1 is the number of incident directions. $\sigma_{V}^{2}$ is the power of noise. Then the covariance matrix of the received signal data and its reference data can be simplified to

(5) $$R_{xx}^{\left(G\right)}=E\left\{x\left(n\right)x^{H}\left(n-jG\right)\right\}={\mathrm{AP}}_{PRNs}A^{H}$$

It can be seen from Equation (5) that the noise component is removed from the covariance matrix. Consider that $R_{xx}$ and $R_{xx}^{\left(G\right)}$ are unavailable in practice, which are usually replaced by the sample covariance matrix

(6) $${\hat{R}}_{xx}\approx \frac{1}{N}X_{N}X_{N}^{H}$$

(7) $${\hat{R}}_{xx}^{\left(G\right)}\approx \frac{1}{N}X_{N}X_{Nref}^{H}$$

where $X_{N}=\left[x\left(n\right)\ldots x\left(n-\left(N-1\right)\right)\right]$ and $X_{Nref}=\left[x\left(n-jG\right)\ldots x\left(n-\left(N-1\right)-jG\right)\right]$, *N* denotes the data block length. In order to improve the accuracy of covariance matrix construction and DOA estimation, we fully exploit the redundancy of the C/A-code, multiple data blocks, and their corresponding reference data blocks are used in this paper, which leads to lowered estimation covariance and, hence, improved DOA estimation performance [26]. In addition, the DOA estimation performance can be improved as the number of samples increases [3]. However, increasing the number of data blocks and samples means higher computational complexity. In this regard, the balance of DOA estimation performance and computational cost should be considered in practical applications.

On the other hand, considering the spoofing continuously adjusts the code phase in the tracking loop to replace the genuine signal tracking point, at the same time, the signal strength of the spoofing signal is gradually increased. In the process of adjusting the code phase and power of the spoofing, the rank of the signal subspace will be reduced due to the enhanced coherence. Therefore, the DOAs of multiple signal sources can hardly be identified.

The Spatial Smooth MUSIC (SSMUSIC) technique [27] is a good candidate to reduce the correlation between multiple sources, which can improve the accuracy of DOA estimation. Specifically, we first divide the ULA with *M* array elements into *p* overlapping subarrays, where each subarray contains m=M−p+1 sensors, and the *k*-th subarray, its corresponding received signal vector $x^{k}\left(n\right)$ can be expressed as:

(8) $$x^{k}\left(n\right)=Z_{k}x\left(n\right)$$

where $Z_{k}=\left[0_{m\times (k-1)}{\left|I\right.}_{m\times m}\left|0_{m\times (p-k)}\right.\right]$, $I_{m\times m}$ represents the *m*-dimensional identity matrix. According to Equation (5), the denoised forward spatial smoothing covariance matrix $R_{f}$ and the denoised backward spatial smoothing convariance matrix $R_{b}$ can be obtained by

(9) $$\begin{array}{cc} R_{f} & =\frac{1}{p}\sum_{k=1}^{p}Z_{k}R_{xx}^{\left(G\right)}Z_{{}_{k}}^{H}=AR_{s}^{f}A^{H} \end{array}$$

(10) $$\begin{array}{cc} R_{b} & =\frac{1}{p}\sum_{k=1}^{p}Q_{k}{\left(R_{xx}^{\left(G\right)}\right)}^{*}Q_{{}_{k}}^{H}=AR_{s}^{b}A^{H} \end{array}$$

where $Q_{k}=\left[0_{m\times (k-1)}\left|J_{m\times m}\right.\left|0_{m\times (p-k)}\right.\right]$, $R_{s}^{f}$ and $R_{s}^{b}$ represent the forward spatial smoothing matrix and the backward spatial smoothing matrix of the signals, respectively. Furthermore, it can be seen $R_{f}$ and $R_{b}$ satisfy

(11) $$R_{b}=J{R_{f}}^{*}J$$

in which, J is the transformation matrix whose back-diagonal elements are 1, which can be described as

(12) $$J={\left[\begin{array}{ccc} 0 & \cdots & 1\\ \vdots & ⋰ & \vdots \\ 1 & \cdots & 0 \end{array}\right]}_{m\times m}^{}$$

Furthermore, the forward/backward spatial smoothing covariance matrix ${R_{f}}_{b}$ based on $R_{b}$ and $R_{f}$ can be formulated as

(13) $${R_{f}}_{b}=\frac{1}{2}\left(R_{b}+R_{f}\right)=\frac{1}{2}A\left(R_{s}^{f}+R_{s}^{b}\right)A^{H}=A{\bar{P}}_{PRNs}A^{H}$$

where ${\bar{P}}_{PRNs}$ is the averaged $P_{PRNs}$. $R_{fb}$ is now full rank, which enables us to identify the DOAs of related sources. As a basic step, the derived covariance matrix $R_{fb}$ will be applied to the subsequent DOA estimation to reduce the influence of the noise and correlation between sources on the DOA estimation accuracy. Furthermore, increasing the number of subarray elements *m* can expand the aperture of each sub-array, thus the degree-of-freedom (DOF) and resolution of DOA estimation can also be enhanced. It is worth noting that in practice, the $R_{xx}^{\left(G\right)}$ in the above equation is replaced by ${\hat{R}}_{xx}^{\left(G\right)}$.

#### 3.1.2. Eigen-Spatial Spectrum Construction

The subspace DOA estimation algorithm, which can break the Rayleigh limit, has been successfully applied to radar, wireless communication, and other fields [28]. Nevertheless, when the method is applied to DOA estimation of spoofing, multipath, and authentic satellite signals, two challenges will be encountered. First, the signal subspace is not fully utilized and the estimation performance will be seriously reduced by the low SNR and related signal sources. Second, it needs to perform eigen decomposition to construct the signal subspace and the noise subspace, which will increase the computational complexity of the algorithm.

To address these challenges, an improved eigen-spatial spectrum is constructed by derived covariance matrix in Equation (13). The proposed algorithm does not need to perform eigen decomposition, and the introduction of the preprocessed covariance matrix $R_{fb}$ and eigenspace can well overcome the model mismatch caused by low SNR and related signals. Specifically, we first partition the steering matrix A by

(14) $$A=\left[\begin{array}{c} A_{1}\\ A_{2} \end{array}\right]$$

in which $T^{H}A_{1}=A_{2}$. **T** is the propagator, $A_{1}$ and $A_{2}$ are composed of the first L+1 and M−L+1 rows of A respectively. Consequently, the preprocessed covariance matrixpreprocessed covariance matrix $R_{fb}$ can be devided into

(15) $$\begin{array}{cc} R_{fb} & =\left[\begin{array}{c} A_{1}\\ A_{2} \end{array}\right]{\bar{P}}_{PRNs}\left[A_{1}\;A_{2}\right]\\ & =\left[\begin{array}{cc} A_{1}{\bar{P}}_{PRNs}A_{1}^{H} & A_{1}{\bar{P}}_{PRNs}A_{2}^{H}\\ A_{2}{\bar{P}}_{PRNs}A_{1}^{H} & A_{2}{\bar{P}}_{PRNs}A_{2}^{H} \end{array}\right]\\ & =\left[\begin{array}{cc} A_{1}{\bar{P}}_{PRNs}A_{1}^{H} & A_{1}{\bar{P}}_{PRNs}A_{1}^{H}T\\ A_{2}{\bar{P}}_{PRNs}A_{1}^{H} & A_{2}{\bar{P}}_{PRNs}A_{1}^{H}T \end{array}\right]\\ & =\left[S\;H\right] \end{array}$$

where S and H are the matrices formed by the first L+1 columns and the last M−L+1 columns of $R_{fb}$, respectively. Since the noise component in $R_{fb}$ has been supressed, the obtained eigen-subspace is composed of signal subspace and null subspace, which is different from traditional eigen-subspace (including noise subspace and signal subspace). In the absence of noise, H and S satisfy

(16) H=ST

After that, we can obtain the propagator $T=S^{+}H$, where $S^{+}$ denotes pseudo-inverse of S. Notably, the accuracy of the estimated **T** is only limited by the sample covariance matrix [29]. Therefore, in order to reduce the influence of **T** and improve the accuracy of subsequent DOA estimation algorithm, multiple data blocks are utilized to get a more accurate sample covariance matrix. Let $U_{0}^{H}=\left[T^{T},-I_{M-L-1}\right]$, it can be seen from Equation (14)

(17) $$U_{0}^{H}A=\left[T^{T},-I_{M-L-1}\right]A=\left[T^{T},-I_{M-L-1}\right]\left[\begin{array}{c} A_{1}\\ A_{2} \end{array}\right]=0$$

Equation (18) demonstrates the subspace $U_{0}$ and the steering matrix A are orthogonal. The resulting DOAs can be estimated by searching the peaks of the novel eigen-spatial spectrum

(18) $$f\left(\theta \right)=\frac{a^{H}\left(\theta \right)R_{{{}_{f}}_{b}}^{+}a\left(\theta \right)}{a^{H}\left(\theta \right)U_{0}U_{0}^{H}a\left(\theta \right)}$$

When $\theta \;=\;\theta_{i}\left(i=1,2,\cdots L+1\right)$, $a^{H}\left(\theta \right)U_{0}U_{0}^{H}a\left(\theta \right)=0$. The position of the *i*-th peak $f\left(\theta_{i}\right)$ in the spatial spectrum represents the DOA of the *i*-th source. It is worth noting that the proposed DOA estimation algorithm makes full use of the derived covariance matrix $R_{fb}$ and its corresponding subspace $U_{0}$, which is more robust to the impact of related signals and low SNR. Besides, it is distinct from the weighted MUSIC algorithm that the proposed DOA estimator is a ratio of the two quadratic forms $a^{H}\left(\theta \right)R_{{{}_{f}}_{b}}^{+}a\left(\theta \right)$ and $a^{H}\left(\theta \right)U_{0}U_{0}^{H}a\left(\theta \right)$ [30].

### 3.2. GNSS Spoofing Detection and Mitigation

In this section, we propose a reliable method for GNSS spoofing detection and mitigation. An optimization problem is firstly formulated for power estimation. Then, the power comparison and cross-correlations are combined to detect the spoofing and multipath, which makes full use of the spatial and temporal characteristics, where each individual signal is separated from others by oblique projections. Finally, we provide an interference mitigation scheme based on subspace oblique projection and beamforming. Thus, the authentic signals can be enhanced while spoofing and multipath are suppressed.

#### 3.2.1. Enhanced Power Estimation

Considering we have obtained the high-resolution estimated DOAs of *K* incident sources, thus we devise an optimization problem for power estimation based on the denoised sample covariance matrix ${\hat{R}}_{xx}^{\left(G\right)}$, where the estimated DOA ${\hat{\theta }}_{k}$ can be used as a priori information. Specifically, the source power is estimated by matching ${\hat{R}}_{xx}^{\left(G\right)}$ and corresponding theoretical covariance matrix. Intuitively, the optimization problem can be formulated as

(19) $$\begin{array}{cc} \; & \min_{\bar{p}\left({\hat{\theta }}_{k}\right)}{∥{\hat{R}}_{xx}^{\left(G\right)}-A\left({\hat{\theta }}_{k}\right)P_{PRNs}\left({\hat{\theta }}_{k}\right)A{\left({\hat{\theta }}_{k}\right)}^{H}∥}_{F}^{2}\\ & \mathrm{subject}\;\mathrm{to}\;\bar{p}\left({\hat{\theta }}_{k}\right)\geq 0 \end{array}$$

where $\bar{p}\left({\hat{\theta }}_{k}\right)=\;$ diag $\left(P_{PRNs}\left({\hat{\theta }}_{k}\right)\right)$ denotes the estimated power of each PRN signal at source angle ${\hat{\theta }}_{k}$. ${∥∥}_{F}$ represents the Frobenius norm. The above optimization problem is considered as a typical inequality constrained least squares problem, whose solution can be given by

(20) $$\bar{p}\left({\hat{\theta }}_{k}\right)\;=[B^{H}B{]}^{-1}B^{H}v$$

where

(21) $$B=[vec\left(a\left({\hat{\theta }}_{1}\right)a^{H}\left({\hat{\theta }}_{1}\right)\right),vec\left(a\left({\hat{\theta }}_{2}\right)a^{H}\left({\hat{\theta }}_{2}\right)\right),\ldots vec\left(a\left({\hat{\theta }}_{K}\right)a^{H}\left({\hat{\theta }}_{K}\right)\right)]$$

And

(22) $$v=vec({\hat{R}}_{xx}^{\left(G\right)})$$

where vec(·) represents the vectorization operation of the matrix.

The proposed source estimation algorithm, which makes full use of the denoised sample covariance matrix ${\hat{R}}_{xx}^{\left(G\right)}$, can achieve high-resolution DOA estimation and simultaneously obtain the enhanced power estimation. As the characteristic parameters for source estimation, DOA and power will be used to GNSS spoofing detection in the next subsection.

#### 3.2.2. The Combined Spoofing Detection Technology

The combined spoofing detection technology is based on power comparison and cross-correlation peaks monitoring, both of which rely on high-precision DOA estimation. Specifically, since the oblique projection operation extracts the target signal by projecting the measured data along the direction of the target signal subspace without affecting the data in the oblique subspace, the oblique subspace projection technique is first used to separate all signal sources and divide sources into *K* spatial channels according to the estimated DOAs. Define $E_{\Theta_{C}\Theta_{D}}$ as the oblique projection matrix with range space $\langle \Theta_{C}\rangle$ and null space $\langle \Theta_{D}\rangle$, which can be formulated as

(23) $$E_{\Theta_{C}\Theta_{D}}=\Theta_{C}{\left(\Theta_{C}^{H}P_{\Theta_{D}}^{⊥}\Theta_{C}\right)}^{-1}\Theta_{C}^{H}P_{\Theta_{D}}^{⊥}$$

where $P_{\Theta_{D}}^{⊥}$ represents the orthogonal projection matrix of $\langle \Theta_{D}\rangle$. Furthermore, the spoofing $z_{sp}\left(n\right)$, the genuine satellite signal $z_{au}^{i}\left(n\right)$ and the multipath $z_{mu}^{q}\left(n\right)$ can be expressed as

(24) $$\begin{array}{c} \;z_{sp}\left(n\right)=E_{a\left(\theta_{s}\right)\left(A∼a\left(\theta_{s}\right)\right)}x\left(n\right)\\ \;z_{au}^{i}\left(n\right)=E_{a\left(\theta_{i}\right)\left(A∼a\left(\theta_{i}\right)\right)}x\left(n\right)\\ z_{mu}^{q}\left(n\right)=E_{a\left(\theta_{q}\right)\left(A∼a\left(\theta_{q}\right)\right)}x\left(n\right) \end{array}$$

where $\theta_{s}$, $\theta_{i}\left(i=1,2,\cdots N_{a}\right)$ and $\theta_{q}\left(q=1,2,\cdots N_{m}\right)$ represent the spatial incidence angles of spoofing, multipath, and genuine satellite signal, respectively, which can be obtained by Equation (18). $K=N_{a}+N_{m}+1$ is the total number of DOAs for spoofing, multipath and genuine satellite signals. $A∼a\left(\theta_{i}\right)=\left[a\left(\theta_{1}\right)\ldots a\left(\theta_{i-1}\right),a\left(\theta_{i+1}\right)\ldots a\left(\theta_{L}\right),a\left(\theta_{s}\right)\right]$, $A∼a\left(\theta_{q}\right)=\left[a\left(\theta_{1}\right)\ldots a\left(\theta_{q-1}\right),a\left(\theta_{q+1}\right)\ldots a\left(\theta_{L}\right),a\left(\theta_{s}\right)\right]$ and $A∼a\left(\theta_{s}\right)=\left[a\left(\theta_{1}\right),a\left(\theta_{2}\right),\ldots a\left(\theta_{L}\right)\right]$.

Then, we perform cross-correlation operations on the signals of the *K* spatial channels, where the cross-correlation results of the *k*-th channel and the *p*-th channel can be can be obtained by

(25) $$R_{cross}=IFFT\left\{FFT\left(z^{k}\right)•{\left(FFT\left(z^{p}\right)\right)}^{*}\right\}$$

where p,k=1,2,3·⋯K and p≠k. $z^{k}$ and $z^{p}$ are the signal vectors of the *k*-th spatial channel and the *p*-th spatial channel, respectively.

In terms of power comparison, in order to successfully take over the target receiver, the power of the spoofing is usually greater than the genuine signals, especially when the spoofing is aligned with the satellite signal code phase, thus the strongest power signal is first suspected to be the spoofing. Furthermore, in general, the power of the multipath is lower than satellite signal. In this regard, the spoofing and multipath can be preliminarily distinguished by accurate power calculation and comparison.

From the perspective of cross-correlation operation, under the assumption that multiple spoofing signals are transmited by a single antenna, the spatial direction corresponding to the spoofing must contain multiple counterfeit PRN signals. Hence, there will be multiple cross-correlation peaks in the cross-correlation results between spoofing and other signals, which is the property that multipath signals do not have. It is because the multipath is only the attenuation of a specific PRN signal and there are no multiple cross-correlation peaks between it and other authentic signals.

In particular, for the low-power spoofing, although the reliability of power comparison results may be affected by the DOA estimation and power estimation error, there must be multiple cross-correlation peaks in cross-correlation results between it and other signals. On the contrary, if there is no spoofing, there will be no significant difference in power between the spatial signals, and due to the orthogonality of the PRN codes, there will be no multiple cross-correlation peaks between the signals. As for the multipath corresponding to high-power satellite signal, even if its power level is equivalent to other satellite signals, the spatial channel where it is located will only have a cross-correlation peak with the specific PRN signal. Therefore, the combination of power comparison and cross-correlation peak monitoring provides an effective approach to distinguish spoofing from multipath and genuine signals.

In order to explain the decision-making process of the detection algorithm more clearly, we provide Table 1, which gives the output results corresponding to different situations.

#### 3.2.3. Interference Mitigation

Without loss of generality, we adopt $H_{i}$ to represent the result of spoofing detection, where $H_{0}$ indicates the interference-free, and  $H_{i}$, i=1,2,3 correspond respectively to multipath, spoofing, and coexistence of spoofing and multipath. Since the DOAs of spoofing, multipath, and authentic signals can be obtained after the spoofing detection module, we formulate the following interference suppression method based on the results of spoofing detection.

(1) $H_{4}$ is true:

Once $H_{4}$ is true, according to Equation (24), the subspace oblique projection is utilized to suppress the spoofing and multipath, which can be expressed as   

(26) $$z\left(n\right)=\left(I-E_{a\left(\theta_{s}\right)\left(A∼a\left(\theta_{s}\right)\right)}-E_{a\left(\theta_{q}\right)\left(A∼a\left(\theta_{q}\right)\right)}\right)x\left(n\right)$$

where $q=1,2,\cdots N_{m}$. In addition, in order to maximize the power of desired satellite signal, we beamform each satellite signal by designing the weight vector, which can be obtained by

(27) $${w_{i}}^{H}=a^{H}{\left(\theta_{i}\right)}^{},i=1,2,\cdots N_{a}$$

Therefore, the final output of the proposed anti-spoofing scheme is given by:

(28) $$y_{i}\left(n\right)={w_{i}}^{H}z\left(n\right)$$

(2) $H_{3}$ is true:

Since the spoofing can be eliminated by subspace oblique projection, thus we first get the oblique projection

(29) $$\Omega =I-E_{a\left(\theta_{s}\right)\left(A∼a\left(\theta_{s}\right)\right)}$$

After that, the  weight vector ${w_{i}}^{H}$ is utilized to reduce attenuation of authentic satellite signals, thus the final output

(30) $$y_{i}\left(n\right)={w_{i}}^{H}\Omega x\left(n\right)$$

(3) $H_{2}$ is true:

Similar to the method in $H_{3}$, we maximize the power of genuine signals through beamforming while mitigating the multipath, the final output signals can be calculated by

(31) $$y_{i}\left(n\right)={w_{i}}^{H}\left(I-E_{a\left(\theta_{q}\right)\left(A∼a\left(\theta_{q}\right)\right)}\right)x\left(n\right)$$

(4) $H_{0}$ is true:

In this case, interference mitigation no longer needs to be performed. The output, which maximizes the true signal power, can be described as

(32) $$y_{i}\left(n\right)={w_{i}}^{H}x\left(n\right)$$

### 3.3. Overall Spoofing Detection and Mitigation Scheme

In order to summarize the proposed anti-spoofing scheme, all steps are described in Algorithm 1.
**Algorithm 1:** GNSS Anti-Spoofing Scheme  **DOA Estimation**  **Input: **
x(n)   1: Construct the covariance matrix by Equation (5) to suppress the noise component.   2: Reduce the correlation between sources according to Equation (13).
   3: Estimate the DOAs for sources through Equation (18).   **Output:** Estimated DOAs   **Spoofing Detection and Mitigation**
  **Input:** Estimated DOAs    1: The enhanced power estimation is performed by Equation (19).    2: Separate the incident signals according to Equation (24).    3: Obtain the cross-correlation results by Equation (25).    4: Make decisions based on power comparison and cross-correlation results.     5: Calculate the output signal according to the decision results. 

## 4. Simulation Results

Performance of the proposed anti-spoofing method was evaluated through simulations. These include: (a) evaluation of DOA and power estimation; (b) verification of spoofing detection and mitigation in three scenarios. In our simulations, we assume all signals and the sensor array are on the same plane, and the array used by the receiver is a ULA with M=16 sensors, where the distance between sensors is half the carrier wavelength. The additive noise is considered as a zero-mean white Gaussian random process. The number of sources is a priori, which can be obtained by Minimum Description Length ( MDL) [3].

### 4.1. DOA and Power Estimation

In this subsection, we focus on the performance of the proposed DOA and power estimation algorithm. Assuming there are *K* = 2 correlated sources that share the same C/A code come from −10${}^{\circ }$ and 30${}^{\circ }$ direction respectively with the power $p_{1}=p_{2}=1\;W$, both of which have the repetitive properties. Furthermore, *N* = 800 samples are collected in both the data and reference blocks. *L* = 1000 Monte-Carlo trials are performed in each simulation.

#### 4.1.1. DOA Estimation Verification

We now compare the accuracy of the proposed DOA estimation algorithm with the spatial smoothing MUSIC(SS-MUSIC) [27] and eigen space modified MUSIC(ES-MMUSIC) [25] that can be used for correlated sources. Since the SS-MUSIC is not available under low SNR, in order to make a fair comparison, we have also performed noise reduction processing on it. The root mean square error (RMSE) is used to evaluate the performance of DOA estimation, which can be expressed as

(33) $$RMSE=\sqrt{\frac{1}{LK}\sum_{l=1}^{L}\sum_{k=1}^{K}{\left({\hat{\theta }}_{k,l}-\theta_{k}\right)}^{2}}$$

in which ${\hat{\theta }}_{k,l}$ and $\theta_{k}$ denote the *k*-th estimated DOA in the *l*-th Monte Carlo trial and the *k*-th setting DOA respectively.

**Figure 2:**
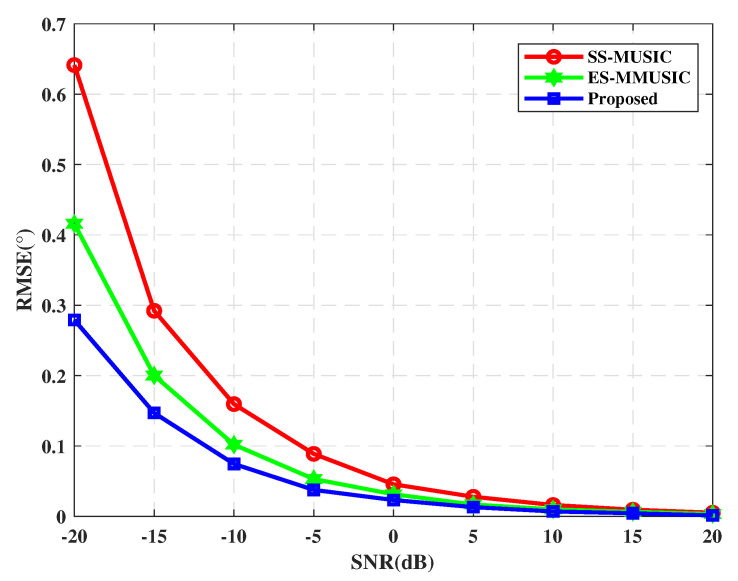
The root mean square error (RMSE) of direction of arrival (DOA) estimation versus SNR.

The RMSE of the DOA estimation versus the SNR is shown in Figure 2. Table 1 shows the running time of the three algorithms. It should be pointed out that the ES-MMUSIC algorithm can only perform effective decorrelation processing on two correlated sources [31], hence two correlated sources are selected for this simulation. On the contrary, the proposed algorithm and SS-MUSIC can process multiple correlated sources, as long as the number of sub-array elements *m* is greater than the number of sources. In our simulation, subarray contains *m* = 12 sensors.

In Figure 2, we vary the SNR from −20 dB to 20 dB in steps of −5 dB. As shown in Figure 2, the proposed algorithm shows a significant advantage over the other two algorithms, especially under the low SNR, which indicates the accuracy of the proposed approach for correlated sources is improved at low SNR. Obviously, owing to the signal preprocessing and eigen-spatial spectrum construction, the proposed DOA estimator is more robust to the effects of subspace mismatches due to correlated signals and low SNR. Since the signal model established in the Section 2 belongs to the related source and the SNR of the signal is very low, the proposed algorithm is beneficial for the DOA estimation of genuine signals, multipath, and spoofing. In addition, it can be seen from Table 2 that the running time is not much different between the proposed algorithm and ES-MMUSIC, and both of which are less than SS-MUSIC. Although the proposed algorithm still needs to divide the array into subarrays, the eigen decomposition is avoided by introducing PM, which can reduce the amount of calculation to a certain extent.

#### 4.1.2. Power Estimation Performance

In this experiment, the performance of the power calculated by Equation (19) is analyzed, where the RMSE of the power estimation is utilized to evaluate the performance, which can be defined as

(34) $$RMSE_{p}=\sqrt{\frac{1}{LK}\sum_{l=1}^{L}\sum_{k=1}^{K}{\left({\hat{p}}_{k,l}-p_{k}\right)}^{2}}$$

in which ${\hat{p}}_{k,l}$ represents the estimated power of the *k*-th signal in the *l*-th Monte Carlo trial and $p_{k}$ is the perfect power value.

**Figure 3:**
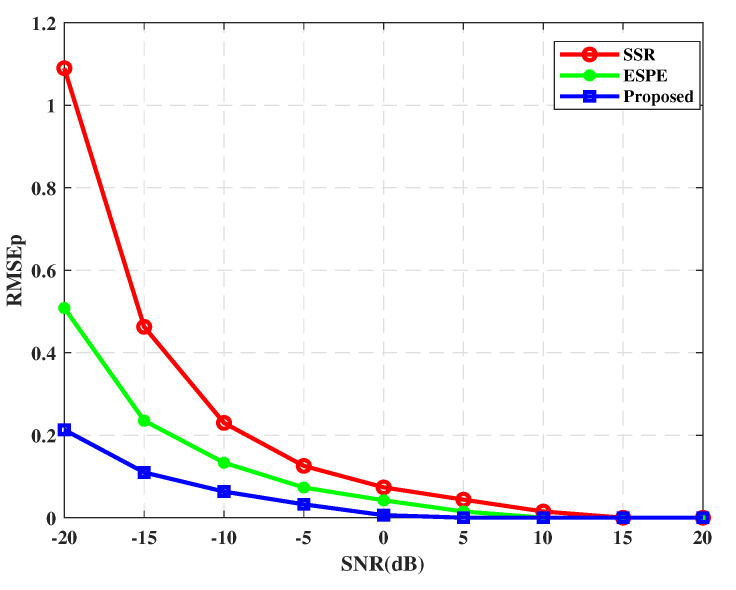
The RMSE of power estimation versus SNR.

We compare the power estimation performance of the proposed algorithm with the sparse signal reconstruction (SSR) algorithm in [32] and the method in [25]. Since the power estimation method in [25] is based on the eigen space, for simplicity, we use the abbreviation ESPE to denote it in this paper. The RMSE of power estimation versus SNR is displayed in Figure 3. We can observe from Figure 3 that the accuracy of the proposed power estimation algorithm can be improved efficiently as the SNR increases. Furthermore, the proposed algorithm outperforms the SSR algorithm and ESPE algorithm in the whole SNR range we considered. Even when the SNR is as low as −20 dB, the power estimation accuracy is also satisfactory. Consequently, the enhanced power estimation can be performed by the proposed optimization problem.

### 4.2. Spoofing Detection and Mitigation

In this subsection, more simulation results have been provided to prove the performance of the proposed GNSS spoofing detection and mitigation method in different application scenarios. To obtain time and three-dimensional position coordinates, four genuine satellite signals are considered in our simulation. The signal received by the antenna array is down-converted to an intermediate frequency of 4.092 MHz, and the sampling frequency is 37.85 MHz. Each C/A code chip has 37 sampling points, and the corresponding spreading gain is G=1023×37. The sampling point length of the data block and reference data block used in this simulation is *N* = 37,000, and the data length of 20 ms is selected for simulation. In order to improve the estimation accuracy of the covariance matrix, 7 pairs of data blocks $X_{N}$ and $X_{Nref}$ are used. In the simulation, it is assumed that spoofing and multipath are aligned with the code phase of the satellite signal within 6 samples (0.16 chips) and 13 samples (0.35 chips), respectively. The power of the satellite signals at the receiver is −160 dBW. Furthermore, the SNR of the satellite signal at the receiver is −20 dB while the SNR of the spoofing and multipath varies according to different scenarios. For each simulation scenario, 1000 Monte-Carlo trials are performed for power estimation. The following is divided into three different scenarios for simulation verification.

**Figure 4:**
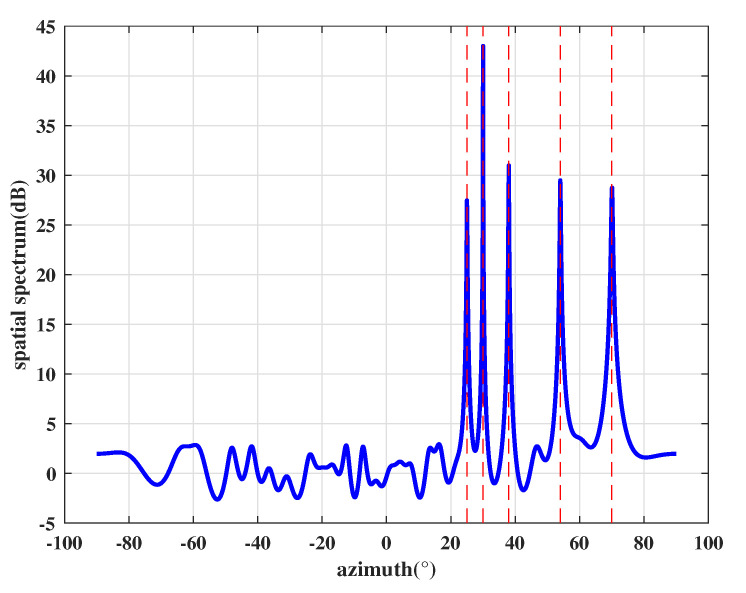
Spatial spectrum estimation results.

- Scenario 1:

In the first experiment, we consider four genuine signals PRN1, PRN6, PRN9, and PRN26 that are transmitted from 25${}^{\circ }$, 38${}^{\circ }$, 54${}^{\circ }$, and 70${}^{\circ }$, respectively. It is assumed that there is one spoofing that comes from 30${}^{\circ }$, which contains four counterfeit signals with the same PRNs as the four genuine satellite signals. The power of each spoofing signal is 3 dB larger than its corresponding genuine signals. According to the proposed method, we first perform DOA estimation and the obtained spatial spectrum is shown in Figure 4. The red dashed lines indicate the DOAs of incident sources. Figure 4 illustrates that the proposed DOA estimation based on preprocessing and improved eigen space can accurately estimate the incident direction of all signals.

**Figure 5:**
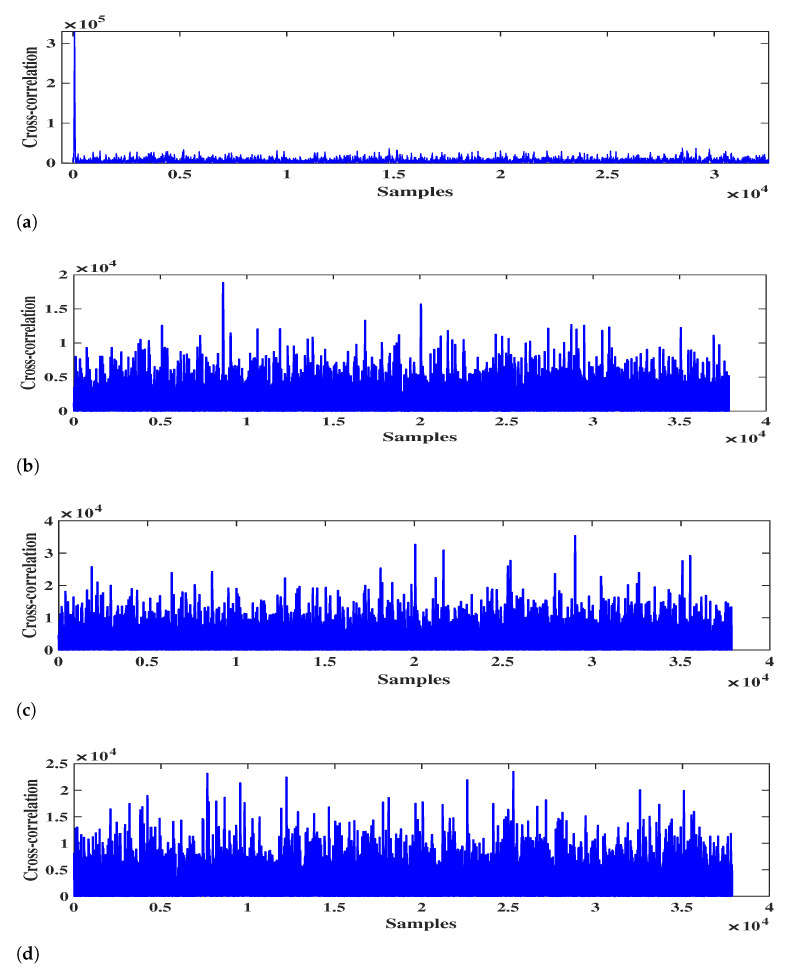
The cross-correlation results. (**a**) 25${}^{\circ }$ and 30${}^{\circ }$; (**b**) 25${}^{\circ }$ and 38${}^{\circ }$; (**c**) 25${}^{\circ }$ and 54${}^{\circ }$; (**d**) 25${}^{\circ }$ and 38${}^{\circ }$.

After that, the power is estimated by Equation (19), where the Equation (24) is used to separate received signals. For simplicity, we use SNR to represent the power of each signal. The estimated SNR corresponding to each DOA is shown in Table 3, which is arranged in descending order. Then, we perform cross-correlation operations on any two spatial signals. Taking the spatial signal with 25${}^{\circ }$ direction as an example, the cross-correlation results between it and 30${}^{\circ }$ signal, 38${}^{\circ }$ signal, 54${}^{\circ }$ signal, 70${}^{\circ }$ signal are shown in Figure 5. It can be seen from Figure 5 that the cross-correlation peak only exists between the 25${}^{\circ }$ signal and 30${}^{\circ }$ signal. Similarly, we perform cross-correlation operation on the remaining signals and count all cross-correlation peaks in Table 4.

It can be seen from Table 3 that the power difference between the strongest #1 signal and the average of #2, #3, #4 and #5 signals is as high as 5.65 dB. According to the proposed method, the DOA corresponding to #1 signal with the highest power is assumed to be the direction of the spoofer. In addition, it can be seen from Table 4 that there are four correlation peaks in the cross-correlation results between #1 signal and other signals. Therefore, it is obvious that the strongest signal has the same PRN code as the other four signals, which indicates that the signal in 30${}^{\circ }$ direction is the spoofing.

**Figure 6:**
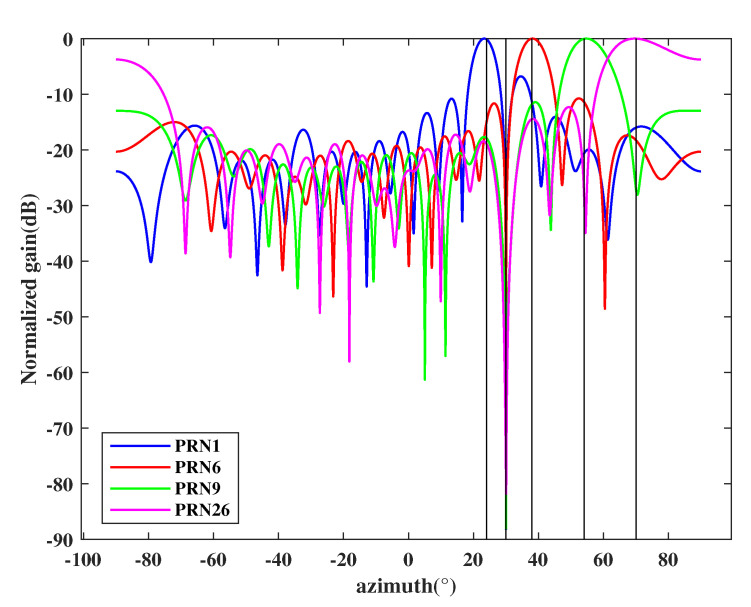
Beam pattern for each authentic satellite.

Based on the above results, the output signals can be calculated by Equation (30) and the final beam pattern of the array is shown in Figure 6. It shows that the proposed interfere mitigation technology can form deep null steering in the DOA of the spoofing while obtaining the maximum gain of satellite signals.

- Scenario 2:

In the second example, in order to verify the effectiveness of the proposed method in multipath environment we consider four authentic signals PRN1, PRN6, PRN9, and PRN26 coming from −50${}^{\circ }$−30${}^{\circ }$, 0${}^{\circ }$ and 20${}^{\circ }$ direction. Furthermore, one multipath signal, which pertains to PRN6, comes from 50${}^{\circ }$ direction. In this simulation, the SNR of satellite signal PRN6 is 1 dB higher than other satellites, and the multipath signal has the same power as the other signals.

**Figure 7:**
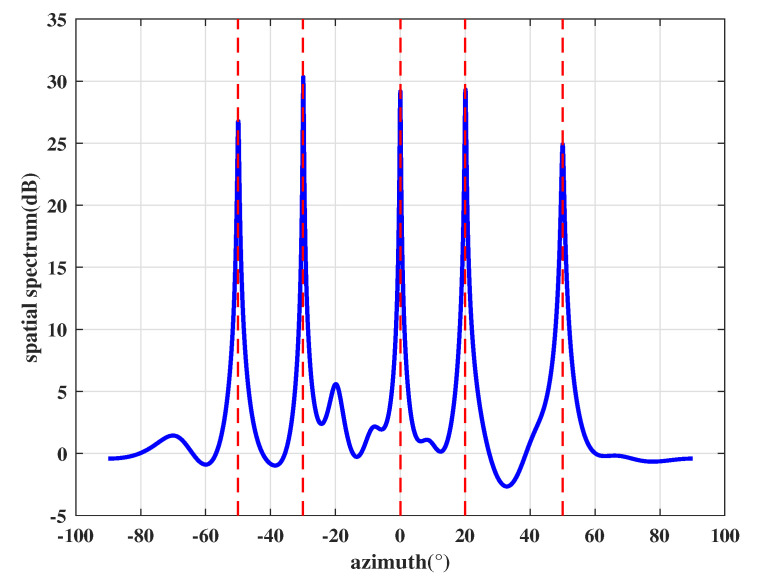
Spatial spectrum estimation results.

The estimated spatial spectrum is shown in Figure 7, which shows the proposed DOA estimation algorithm can effectively obtain the directions of all signals (including authentic signals and multipath). After that, we estimate the power of each signal and sort them in descending order, as shown in the Table 5. Then, the cross-correlation results of all signals are shown in Table 6.

As Table 5 and Table 6 show, the strongest #1 signal is only 1.26 dB higher than the average of the other signals and there is only one correlation peak in the cross-correlation results between #1 and #5. Therefore, according to the proposed anti-spoofing method, the strongest signal cannot be the spoofing. Furthermore, there is a cross-correlation peak between #1 signal and #5 signal, which is the feature that two authentic signals do not have. Since the multipath signal is the attenuation of genuine signals, the power of multipath is less than its corresponding authentic satellite signal. Based on the above analysis, it can be seen that the #1 signal is the genuine signal while #5 signal is a multipath whose direction is 50${}^{\circ }$.

It is worth noting that this case assumes the multipath and the other three authentic signals have the same power, which is not typical in actual situations. Even in this environment, the proposed method can detect that there is only multipath interference but no spoofing signal, which shows that strong multipath will not affect the accuracy of the spoofing detection. Accordingly, the monitoring of the number of cross-correlation peaks can minimize the impact of power estimation errors on the spoofing detection results.

**Figure 8:**
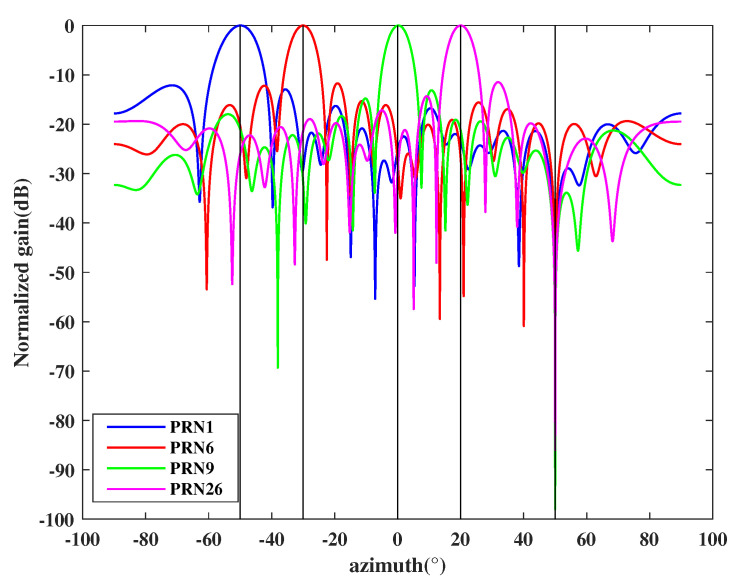
Beam pattern for each authentic satellite.

According to the above results, the final signal vector can be calculated by Equation (31) and the beam pattern of the array in this case is shown in Figure 8. It shows that the proposed anti-spoofing technology can form deep null steering in the DOA of the multipath. Since the beamforming for each satellite is performed, the authentic signals can also get the maximum gain.

**Figure 9:**
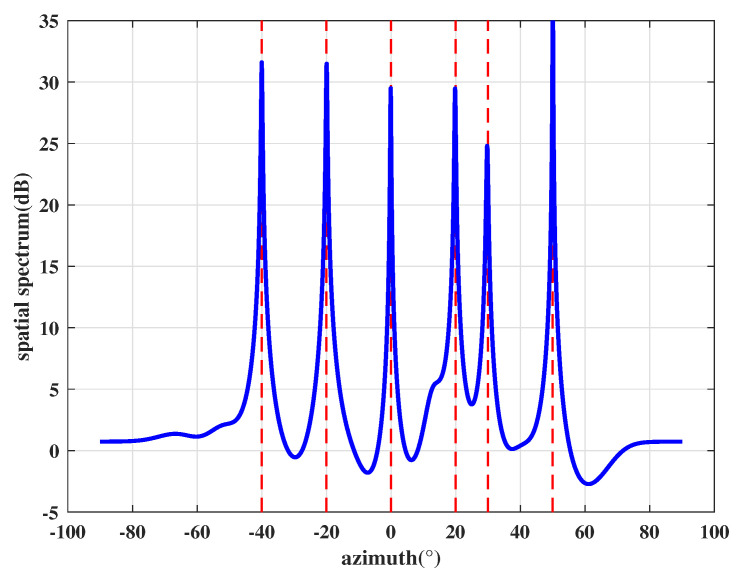
Spatial spectrum estimation results.

- Scenario 3:

Final simulation example considers one spoofing, one multipath, and four authentic signals (PRN1, PRN6, PRN9, and PRN26), where the multipath signal pertains PRN1 and the spoofing transmits four spurious signals that have the same PRNs with the authentic signals. We assume each spoofing is only 0.4 dB higher than the authentic signals [16], and the multipath signal is 1 dB lower than the authentic signals. In this simulation, the DOAs of the PRN1, PRN6, PRN9, and PRN26 are −40${}^{\circ }$, −20${}^{\circ }$, 0${}^{\circ }$, and 20${}^{\circ }$ respectively. The spoofing and the multipath are incident from 50${}^{\circ }$ and 30${}^{\circ }$, respectively. The estimated spatial spectrum is shown in Figure 9 and based on which the estimated DOA can be obtained. Then we estimate the source power corresponding to all spatial DOAs and arrange them in descending order as shown in Table 7. After that, all signals are used to perform cross-correlation, and the result is shown in Table 8.

We can see from Table 7 and Table 8 that the strongest signal and other signals have correlation peaks. The power difference between it and the average value of other signals is 3.04 dB, and the existence of multiple correlation peaks indicates that the strongest signal in 50${}^{\circ }$ direction contains the PRN codes of the other signals, which is the feature that multipath and authentic signals do not possess. Thus 50${}^{\circ }$ is the spatial direction of the spoofing. Moreover, it can be seen from Table 8 that the signal in 30${}^{\circ }$ direction not only has correlation peak with the spoofing but also has a cross-correlation peak with the signal in −40${}^{\circ }$. Due to the orthogonality of PRN codes, there is no possibility of cross-correlation peaks between two genuine satellite signals. In terms of power, the signal power in −40${}^{\circ }$ direction is higher than that in 30${}^{\circ }$ direction. Obviously, the signal with 30${}^{\circ }$ direction is a multipath signal. It is illustrated that the proposed algorithm can effectively distinguish spoofing from multipath and satellite signals under the assumption of single antenna transmission, even if the power of each spoofing is not significantly greater than genuine signals.

**Figure 10:**
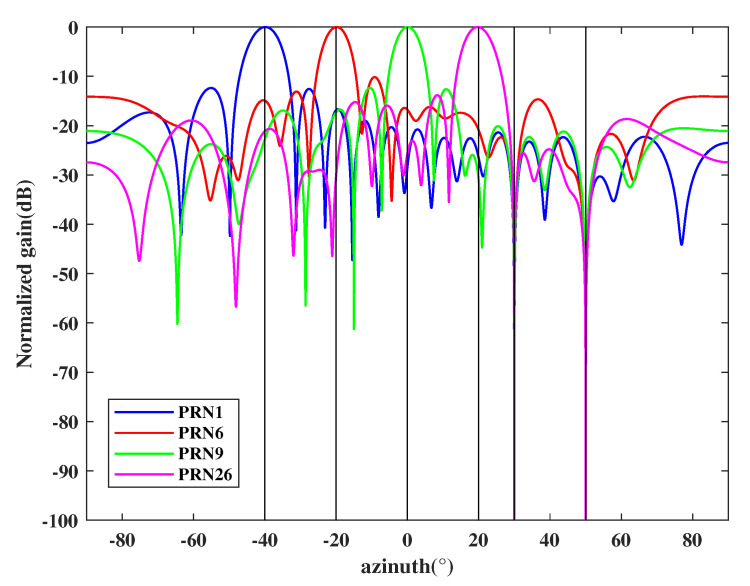
Beam pattern for each authentic satellite.

Since we have obtained the direction of the multipath and spoofing, the subspace oblique projection method in Equation (26) is adopted to suppress them. After that, the final output of each authentic satellite signal can be calculated by Equation (28) and the array beam patterns are shown in Figure 10. It can be seen that the proposed countermeasure can not only form nulls in the direction of multipath and spoofing but also maximize the gain of each authentic satellite signal.

## 5. Conclusions

This paper proposes a spatial-temporal signal processing method based on antenna array to enhance the safety and reliability of GNSS receivers in the presence of spoofing, which can be divided into two stages. In the first stage, the improved eigen space obtained by PM is adopted to perform DOA estimation, in which the self-coherent properties of GNSS signal was fully excavated to remove the noise component before the despreading process. Besides, since the correlation between the GNSS spoofing, multipath, and authentic signals will cause poor DOA estimation performance, we employ the rank restoration technique to reduce the correlation. The second stage dealt with spoofing in different environments, which involved power calculation and cross-correlation peak monitoring. The signal power is formulated by the estimated DOA as a priori information. After that, we perform cross-correlation operation on all signals, and the number of cross-correlation peaks and power are utilized to detect spoofing. Then, the interference suppression method based on subspace oblique projection and beamforming is provided to mitigate spoofing and multipath while enhancing the authentic signals.

Simulation results in Section 4.1 demonstrate the performance advantages of the proposed DOA estimation and power estimation algorithm especially in the case of low SNR and correlated signal sources. In Section 4.2, more simulation results in three scenarios are provided to prove the effectiveness of the proposed anti-spoofing method. It can be seen that the suggested approach can not only accurately distinguish spoofing and multipath but also suppresses them, even in the case of low-power spoofing or high-power multipath. It should be noted that our method aims to distinguish between spoofing and satellite signals based on their differences in the space-time domain. All the operations are not dependent on external hardware and can be readily implemented on the raw digital baseband signal before the despreading of GNSS receivers.

However, in the context of the military application of electronic countermeasures, satellite navigation receiver will be interfered by more and more different spatial distribution interference sources. At this time, the anti-spoofing technology based on ULA still has the problem of insufficient freedom in practical application. In addition, the proposed preprocessing framework can effectively reduce the correlation between authentic signals, spoofing, and multipath, but the effective aperture of the array is also lost. Therefore, as far as the current technology is concerned, how to improve the array antenna structure and increase the freedom of spoofing detection for the actual application scenarios of GNSS will be further investigated.

## Figures and Tables

**Table 1:** Detection decision according to the different detection results.

Detection Results	Decision
Power difference are insignificant; No cross-correlation peaks.	No interference
Only one cross-correlation peaks between two certain signals	Only multipath
Multiple correlation peaks between the signal with highest power and others	Only spoofing
Multiple correlation peaks; One correlation peaks between two certain signals.	Spoofing and multipath

**Table 2:** The DOA estimation operating time(s).

SS-MUSIC	ES-MMUSIC	Proposed
0.742	0.450	0.442

**Table 3:** The estimated SNR of all signals.

	#1	#2	#3	#4	#5
DOA (${}^{\circ }$)	30	54	25	38	70
SNR (dB)	−14.48	−19.96	−20.06	−20.18	−20.33

**Table 4:** The cross-correlation results between different spatial signals.

DOA (${}^{\circ }$)	25	30	38	54	70
25	∼	✓	×	×	×
30	✓	∼	✓	✓	✓
38	×	✓	∼	×	×
54	×	✓	×	∼	×
70	×	✓	×	×	∼

✓ denotes correlation peak exists; × denotes there is no correlation peak; ∼ denotes cross-correlation is not performed.

**Table 5:** The estimated SNR of all signals.

	#1	#2	#3	#4	#5
DOA (${}^{\circ }$)	−30	20	0	−50	50
SNR (dB)	−18.88	−19.96	−20.04	−20.18	−20.37

**Table 6:** The cross-correlation results between different spatial signals.

DOA (${}^{\circ }$)	−50	−30	0	20	50
−50	∼	×	×	×	×
−30	×	∼	×	×	✓
0	×	×	∼	×	×
20	×	×	×	∼	×
50	×	✓	×	×	∼

✓ denotes correlation peak exists; × denotes there is no correlation peak; ∼ denotes cross-correlation is not performed.

**Table 7:** The estimated SNR of all signals.

	#1	#2	#3	#4	#5	#6
DOA (${}^{\circ }$)	50	−40	−20	20	0	30
SNR (dB)	−17.08	−19.78	−19.83	−19.95	−20.13	−20.92

**Table 8:** The cross-correlation results between different spatial signals.

DOA (${}^{\circ }$)	−40	−20	0	20	30	50
−40	∼	×	×	×	✓	✓
−20	×	∼	×	×	×	✓
0	×	×	∼	×	×	✓
20	×	×	×	∼	×	✓
30	✓	×	×	×	∼	✓
50	✓	✓	✓	✓	✓	∼

✓ denotes correlation peak exists; × denotes there is no correlation peak; ∼ denotes cross-correlation is not performed.
